# High-Intensity Focused Ultrasound (HIFU) for Dissolution of Clots in a Rabbit Model of Embolic Stroke

**DOI:** 10.1371/journal.pone.0042311

**Published:** 2012-08-01

**Authors:** Alison Burgess, Yuexi Huang, Adam C. Waspe, Milan Ganguly, David E. Goertz, Kullervo Hynynen

**Affiliations:** 1 Physical Sciences Platform, Sunnybrook Research Institute, Toronto, Ontario, Canada; 2 Department of Medical Biophysics, University of Toronto, Toronto, Ontario, Canada; University of Regensburg, Germany

## Abstract

It is estimated that only 2–6% of patients receive thrombolytic therapy for acute ischemic stroke suggesting that alternative therapies are necessary. In this study, we investigate the potential for high intensity focused ultrasound (HIFU) to initiate thrombolysis in an embolic model of stroke. Iron-loaded blood clots were injected into the middle cerebral artery (MCA) of New Zealand White rabbits, through the internal carotid artery and blockages were confirmed by angiography. MRI was used to localize the iron-loaded clot and target the HIFU beam for treatment. HIFU pulses (1.5 MHz, 1 ms bursts, 1 Hz pulse repetition frequency, 20 s duration) were applied to initiate thrombolysis. Repeat angiograms and histology were used to assess reperfusion and vessel damage. Using 275 W of acoustic power, there was no evidence of reperfusion in post-treatment angiograms of 3 rabbits tested. In a separate group of animals, 415 W of acoustic power was applied and reperfusion was observed in 2 of the 4 (50%) animals treated. In the last group of animals, acoustic power was further increased to 550 W, which led to the reperfusion in 5 of 7 (∼70%) animals tested. Histological analysis confirmed thatthe sonicated vessels remained intact after HIFU treatment. Hemorrhage was detected outside of the sonication site, likely due to the proximity of the target vessel with the base of the rabbit skull. These results demonstrate the feasibility of using HIFU, as a stand-alone method, to cause effective thrombolysis without immediate damage to the targeted vessels. HIFU, combined with imaging modalities used to identify and assess stroke patients, could dramatically reduce the time to achieve flow restoration in patients thereby significantly increasing the number of patients which benefit from thrombolysis treatments.

## Introduction

Systemic delivery of the thrombolytic agent tissue plasminogen activator (tPA), is the primary medical intervention for acute ischemic stroke. tPA is effective at restoring partial flow, depending on thrombus size and time to treatment, leading to approximately 30% improvement in symptoms at 3 months [1]. However, it is estimated that tPA is only administered to 2–6% of stroke patients because of the limited time frame in which it is effective (<4.5 hrs post initial stroke onset) and the increased risk of intracerebral hemorrhage with treatment [1], [2].

Ultrasound is a technique which can improve clot lysis and reduce the amount of tPA required for effective treatment. It has been shown *in vitro* and *in vivo* that ultrasound enhances clot lysis in the presence of a thrombolytic agent compared to the agent alone [3], [4]. Ultrasound has also been combined with systemic delivery of microbubble contrast agents, which improves thrombolysis in the presence or absence of tPA [5]–[8]. It has been demonstrated that ultrasound causes microbubble oscillation leading to effective mechanical disruption of the clot [5]. Ultrasound, microbubbles and tPA together, improve recanalization and have no effect on the hemorrhage rates observed with tPA alone [9], [10].

Although preclinical studies have been optimistic, the results from sonothrombolysis clinical trials have been mixed. In the Combined Lysis of Thrombus in Brain Ischemia Using Transcranial Ultrasound and Systemic t-PA (CLOTBUST) trial, 2 MHz transcranial ultrasound was applied in conjunction with systemic administration of tPA to ischemic stroke patients [11]. In this study, 46% of patients showed improved recanalization rates 2 hrs after treatment, compared to 18% of patients who received tPA alone. Whereas in the TRanscranial low-frequency Ultrasound-Mediated thrombolysis in Brain Ischemia (TRUMBI) trial, unfocused, low frequency (300 kHz) ultrasound was applied using long pulses and was stopped early due to very high hemorrhage rates in patients [12]. These trials indicate further research in the field of sonothrombolysis is necessary.

Recently, the use of high intensity focused ultrasound (HIFU) has been proposed as a stand alone method for clot lysis [13], [14]. It would be advantageous to use HIFU for thrombolysis as this would eliminate the side effects of thrombolytic drugs and potentially reduce the risk of hemorrhage. If successful, HIFU may also reduce treatment time from hours to minutes, which may cause significant reductions in the infarcted brain region and lead to better clinical outcomes. It has recently been demonstrated that HIFU can cause clot lysis within minutes *in vitro* and *in vivo* when used as a stand alone method [13], [14]. Briefly, the ability of HIFU to restore blood flow in occluded femoral arteries *in vivo* was correlated to the creation and subsequent violent collapse of microbubbles. These microbubbles are highly localized in the clot and occur in the absence of systemically injected microbubble contrast agents, suggesting that high-intensities are required for ultrasound to initiate thrombolysis on its own. In the current study, we investigate the feasibility of HIFU for treatment of ischemic stroke in the rabbit brain.

## Materials and Methods

### Animals

All animal procedures were approved by the Sunnybrook Research Institute Animal Care and use Committee and conformed to the guidelines set out by the Canadian Council on Animal Care. 20 male New Zealand White rabbits (2.5–3.0 kg) were obtained from Charles River Laboratories (Sherbrooke, QC). Craniotomies were performed to ensure coupling of the ultrasound wave to the target regions. Animals were anesthetized with a cocktail of ketamine (50 mg/kg) and xylazine (5 mg/kg) and maintained on isoflurane for the duration of the surgery. A micromotor drill (Foredom, Stoelting Co., Wood Dale IL) was used to remove a piece of bone (2×4 cm) from the parietal surface of the skull, creating an acoustic window into a large portion of the brain and leaving the dura mater intact. The skin was sutured back over the window and the wound was allowed to heal for 14 days prior to induction of stroke.

### *In vitro* clot assessment

Clots were prepared based on previous studies [15], [16] and assessed *in vitro*. All clots were formed from arterial rabbit blood, collected in Vacutainer citrate tubes (Becton Dickinson, Mississauga, Canada) and stored at 4° for 24 hrs. In group 1, 200 µL blood was recalcified by the addition of 25 µL CaCl_2_ (100 mmol/L in saline, Sigma Aldrich, St Louis Missouri) and clotted in plastic tubes at 37° for 3 hrs (n = 3). Blood in group 2 was recalcified and clotted by the addition of bovine thrombin (20 µL; 0.2 NIH units/µL, Sigma Aldrich) and stored at room temperature for 3 hrs (n = 3). Clots in groups 3 and 4 were the same as group 2 but stored for 1 hr (n = 3) and 24 hrs (n = 3) respectively. Size, retraction and homogeneity of the clots were qualitatively assessed. A cube (2×2×2 mm) was then cut from each clot and pushed through an injection hub into a 20 g catheter and a saline-filled tube with 1 ml of saline, simulating the *in vivo* embolization procedure. Resulting clots were qualitatively assessed.

### Experimental Stroke Model

Acute experimental strokes were induced in the craniotomized rabbits based on a procedure established by Lapchak and colleagues [15]. Briefly, rabbits were anesthetized using ketamine/xylazine as above. 1 mL of blood was withdrawn from the rabbit's auricular artery immediately after anesthetization in order to prepare emboli. As described above, 200 µL of blood was combined with 20 µl degassed thrombin (0.2 NIH units/µL) and was loaded with 10 µL superparamagnetic iron oxide for clot identification by MRI (1.5×10^7^ particles/mL, Bang's Laboratories Inc, Fischers, IN). The blood clots remained intact at room temperature for 1–3 hrs prior to injection.

The rabbits were then prepared by removing the hair from their head with depilatory cream. The left internal carotid artery of the rabbit was exposed and the external carotid artery was ligated. A 20-gauge plastic catheter, filled with heparinized saline (33 U/mL) was inserted into the common carotid artery, oriented toward the brain. The catheter was secured with ligatures to the common carotid artery and capped with an injection hub. A baseline angiogram of the rabbit brain was obtained using a C-arm x-ray fluoroscopy scanner (OEC, GE Healthcare, Milwaukee, WI). ∼0.5–1.0 mL Omnipaque (350 mg I/mL, GE Healthcare, Milwaukee, WI) was slowly administered through the carotid artery catheter while capturing a series of fluoroscopy images resulting in a baseline angiogram of the rabbit brain vasculature.

The preformed blood clot was then sliced with a straight-edge razor into cubes approximately 2×2×2 mm in size. This size was critical for locating the clot to the middle cerebral artery (MCA). The cube was then suspended in degassed saline and placed in the injection hub. Embolization occurred via flushing the injection hub with 1–2 mL saline. 1 minute following embolization, angiography was repeated to confirm that a blockage had occurred. Subtracted internal carotid angiograms were analyzed and compared to baseline images to confirm a blockage in the left MCA [17]. Immediately after the clot location was confirmed, animals received a bolus injection of heparin (200 U/kg) through the ear vein in order to prevent clot propagation. If a blockage was not observed or if spontaneous recanalization occurred within 15 minutes, a second clot was administered in the same manner. If no blockage was observed on the angiogram following the injection of 3 clots, no further clots were administered and the animal was sacrificed. Spontaneous recanalization was not observed beyond 15 minutes-post injection.

### MRI-guided HIFU

**Figure 1:**
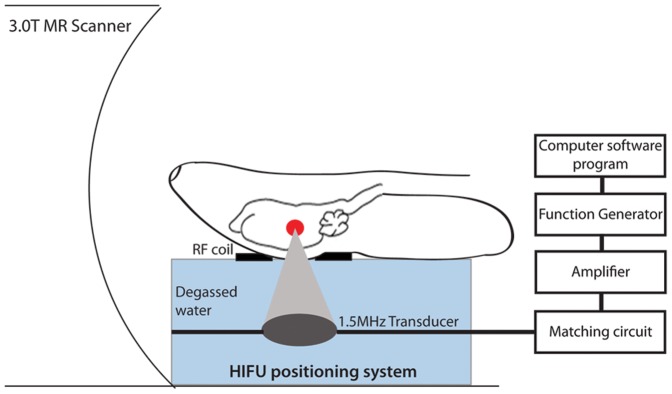
Schematic of experimental procedure. The rabbit is placed supine on a FUS positioning system and the head is coupled to the transducer with degassed water. The entire positioning system is placed inside the 3T MRI. The sonication is controlled by a computer software program which sets the function generator. The signal is amplified before reaching the matching circuit and transducer. Single point sonications are performed every 0.75 mm, moving distal to proximal and superior to inferior, to cover the entire clot surface.

Once a stroke had been confirmed, animals were placed supine on a custom-built HIFU positioning system [18] inside a 3.0T MR scanner (Signa MR750, GE Healthcare, Milwaukee, WI). The rabbit's head was coupled to a degassed water tank to ensure ultrasound wave propagation (Figure 1). Fast gradient echo images (FGRE; TE = 15 ms, TR = 30 ms, slice thickness = 1 mm) are sensitive to iron and were used to detect the clot. Time of flight images (TOF; TE = 4.7 ms, TR = 16 ms, slice thickness = 0.5 mm, matrix = 256×256) were used to confirm blockage in blood flow and to choose precise targeting coordinates for HIFU. Other imaging sequences used include diffusion weighted imaging (TE = 114 ms, TR = 8000), T2*-weighted FGRE imaging (TE = 15 ms, TR = 30 ms), T1-weighted fast spin echo (FSE) imaging (TE = 15 ms, TR = 500 ms) and T2-weighted FSE imaging (TE = 60 ms, TR = 3000 ms). Points for sonications were chosen along all 3 axes. A series of single point sonications were performed every 0.75 mm, moving distal to proximal and superior to inferior. This sonication protocol ensured complete coverage of the blocked vessel. Sonications were initiated within 64±14 min of detecting the blockage in the MCA and each rabbit was only treated with one acoustic power level.

HIFU pulses were generated using a spherically focused air-backed 1.51 MHz transducer (10 cm focal length, 10 cm diameter) and a function generator, (model 33220A, Agilent Technologies, Oswego IL), amplified (model A500, Electronics & Innovation, Rochester NY) and passed through a custom electrical impedance matching circuit. The transducer efficiency, calibrated as described elsewhere [19] was 55% and power levels are expressed in terms of acoustic watts. The sonication parameters used in this study ranged from 275–550 W, 1 ms bursts, 1 Hz pulse repetition frequency and 20 s total exposure duration. The focal spot generated by the transducer was previously measured to be 0.9 mm wide and 7. 1 mm long [13].

Following HIFU treatment, subsequent MR images and fluoro-angiograms were used to determine if recanalization occurred.

### Histological Analysis

Animals were sacrificed with Euthanyl following post-treatment imaging and the brains were removed and fixed in 10% neutral buffered formalin for a minimum of 48 hrs prior to tissue processing and embedding in paraffin wax. 5 serial 4 µm thick sections were taken every 200 µm from a 1 cm block of tissue surrounding the sonicated region. Standard Hematoxylin & Eosin (H&E) staining was performed on one section from each 200 µm tissue block and the following serial section was stained with Prussian blue (Polysciences Inc., Warrington, PA) and counterstained with nuclear fast red to enable the detection of iron. Regions of interest surrounding the MCA from control animals were compared to sections which were sonicated to assess the damage associated with HIFU. CD31 antibodies (Santa Cruz Biotechnology, Santa Cruz CA) were used to label endothelial cells and identify blood vessels. Sections were blocked in 1% donkey serum followed by incubation with the antibody (1∶25) overnight at 4°C. Donkey anti-rabbit biotin secondary antibodies were applied and then developed using DAB peroxidase substrate kit (Vector Labs, Burlinghame CA). High resolution digital images of the tissue sections were captured with a Mirax Scan system (Carl Zeiss MicroImaging, GmbH, Germany) and montages were created using Adobe Photoshop (Adobe Systems Inc., San Jose CA).

### Statistical Analysis

Graphs and analysis was performed using GraphPad Prism 5 (GraphPad Software, San Diego, CA). Reperfusion results were tabulated in a contingency table and analyzed using Fisher's exact test. Significance was noted if p<0.05.

## Results

*In vitro* clot analysis revealed that blood clots formed with thrombin (groups 2, 3) were more robust and less likely to split or deteriorate after being flushed through the catheter than clots formed for 3 hrs at 37° without thrombin. Clots formed for 24 hrs prior to testing were brittle and more likely to break when flushed through the catheter (data not shown). Emboli for injection *in viv*o were prepared with thrombin and maintained for 1–3 hrs prior to injection similar to previous studies [16].

**Figure 2:**
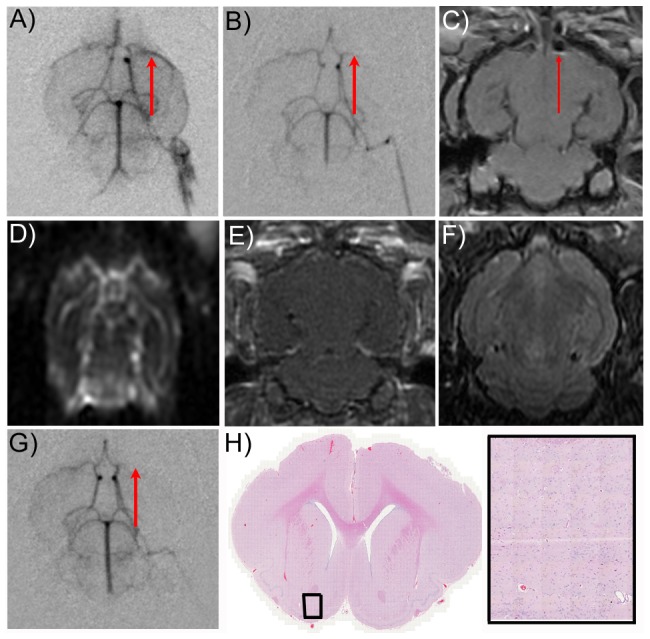
Emboli lodge in the middle cerebral artery. A) Omnipaque contrast agent was administered through the carotid artery while capturing a series of fluoroscopy images resulting in a baseline angiogram of the rabbit brain vasculature. B) Preformed arterial blood clots containing iron nanoparticles and measuring 2 mm^3^ in size were injected through a catheter inserted in the internal carotid artery. Post-stroke angiograms reproducibly located the blockage in the proximal MCA. C) TOF-MR images are used to confirm the location of the clot (red arrow) in the MCA by detection of the iron. X, Y, and Z coordinates were used to align the focal spot of the transducer with the superior distal portion of the clot for sonication. Diffusion weighted imaging (D), contrast enhanced T1 weighted imaging (E) and T2 weighted imaging (F) did not provide significant information and therefore were not used to evaluate the treatment. G) Control animals (no sonication) showed no evidence of reperfusion 1 hr later. H&E analysis of control animals at low (H) and high magnification (I) showed no evidence of tissue damage 1 hr post stroke induction.

Our model of embolic stroke resulted in reproducible blockages in the proximal MCA as detected by C-arm angiography (Figure 2A,B) and confirmed by identification of the iron-loaded clot (hypointense region) on TOF-MRI (Figure 2C). Further characterization using diffusion weighted imaging, contrast enhanced T1 weighted and T2 weighted sequences did not provide substantial information and therefore were not used to assess treatment outcomes (Figure 2D,E,F). 1 hr after stroke induction, control animals were assessed for recanalization using angiography (Figure 2G). No control (non-sonicated) animals showed evidence of reperfusion. H&E staining in control animals at 1 hr demonstrated very little vascular or tissue damage had occurred due to the stroke induction (Figure 2H). We excluded two animals from the study analysis because the clot lodged in the distal, cortical region of the MCA. HIFU treatment was evaluated in 14 rabbits and an additional 6 rabbits were used as non-treated controls. The experimental details are provided in Table 1.

**Table 1:** Summary of experimental details.

Clot #	Elapsed time	Power	Recanalization
1	60 min	N/A	No
1	60 min	N/A	No
2	60 min	N/A	No
1	60 min	N/A	No
2	60 min	N/A	No
1	60 min	N/A	No
2	58 min	275 W	No
1	65 min	275 W	No
1	75 min	275 W	No
2	65 min	415 W	No
1	82 min	415 W	Yes
1	71 min	415 W	No
1	75 min	415 W	Yes
1	68 min	550 W	Yes
3	90 min	550 W	Yes
1	56 min	550 W	No
1	38 min	550 W	Yes
1	57 min	550 W	No
1	39 min	550 W	Yes
1	64 min	550 W	Yes

Each animal included in the study is represented in the table. Clot # refers to the number of clots injected in each animal before stable blockage of the MCA could be confirmed using angiography. The time elapsed between the confirmation of blockage and the HIFU treatment is displayed, followed by the treatment power and treatment outcome.

TOF-MR images were used to choose sonication locations. Single point sonications were performed every 0.75 mm along the length of the clot, moving distal to proximal and superior to inferior. The first set of sonications (1 ms bursts, 0.1% duty cycle, 20 s total duration) were performed using 275 W of acoustic power, which is a power level just below what was previously shown to cause recanalization in an occluded femoral artery model *in vivo* [13]. In 3 animals treated with 275 W, there was no evidence of recanalization on post-treatment angiograms. Similarly, there was no change in TOF- or FGRE-MR images indicating that the HIFU treatment at 275 W was ineffective. In these animals, we analyzed H&E stained sections for bleeding and for ultrasound-induced tissue damage as previously described [20]. There was no evidence of increased bleeding, necrosis or vacuolation within the tissue in the targeted region.

**Figure 3:**
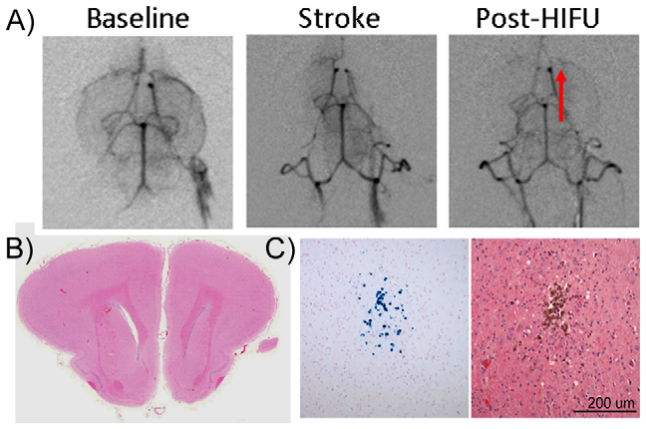
HIFU (415 W) causes partial recanlization in 50% of blocked vessels. A) Recanalization was observed in angiograms taken post-HIFU treatment. 2/4 (50%) of animals treated with 415 W power, exhibited recanalization when compared to post-stroke angiograms. There was no damage or leakage from the vessel apparent on the angiogram. B) Post-treatment H&E showed no evidence of tissue damage or hemorrhage due to HIFU in the region of sonication or in the surrounding brain tissue. Scale bar = 500 µm. C) Prussian blue staining was used to detect small fragments of the disrupted iron-loaded blood clots. In one animal, small fragments were identified in the cortex, downstream of the MCA. Scale bar = 200 µm.

In the second group of animals (n = 4), the acoustic power was increased to 415 W while keeping the other sonication parameters constant. At this power, we observed recanalization on angiograms in 2/4 animals (50%) with no apparent damage or leakage from the vessel (Figure 3A). Post-treatment histology showed no evidence of tissue damage or hemorrhage, specifically due to HIFU in the region of sonication (Figure 3B). In one animal, small fragments of the disrupted iron-loaded blood clots were identified in the cortex following treatment, using Prussian blue staining (Figure 3C).

**Figure 4:**
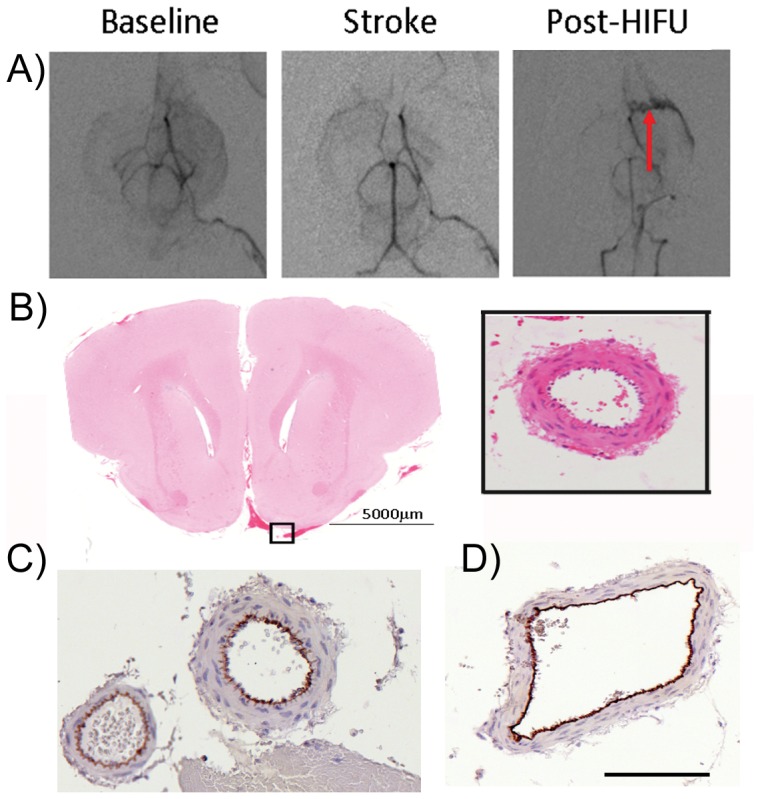
HIFU (550 W) recanalizes ∼70% of blocked vessels with no arterial injury. In most animals treated with 550 W, recanalization, similar to what was achieved with 415 W was observed. A) In one animal, a greater extent of recanalization was achieved but was accompanied by an apparent tortuous MCA vessel suggesting some damage occurred to the MCA during sonication. B) Histological analysis from these animals revealed minor bleeding at the base of the brains; however the MCA appeared intact in these sections (inset). C) CD31 immunohistochemistry labeled endothelial cells and confirmed that the arteries and arterioles are viable in the sonicated regions. Scale bar (B inset, C, D) = 500 µm.

The acoustic power was further increased to 550 W for treatment of the last group of animals (n = 7). Recanalization, similar to what was achieved with 415 W was observed in 5 animals using post-treatment angiograms. In these cases, there was no evidence of bleeding or damage to the vessels on the post-treatment angiograms, nor was there any sign of histological damage as determined by H&E. In 1 of the 5 animals exhibiting reperfusion, a greater extent of recanalization was achieved but was accompanied by some diffuse contrast agent surrounding the MCA (Figure 4A) suggesting some damage occurred to the MCA during sonication. Histological analysis from this animal revealed bleeding at the base of the brain; however, the MCA appeared intact in this section (Figure 4B), suggesting that the bleeding was not coming from the targeted vessel.

CD31 immunohistochemistry was used to detect endothelial cells and assess vessel integrity after treatment. The staining revealed that vessels in the sonicated region were similar in appearance to the control, non-sonicated hemispheres (Figure 4C). In particular, large arteries and arterioles appeared intact and undamaged in the sonicated regions indicating the bleeding was not from the target vessels.

**Figure 5:**
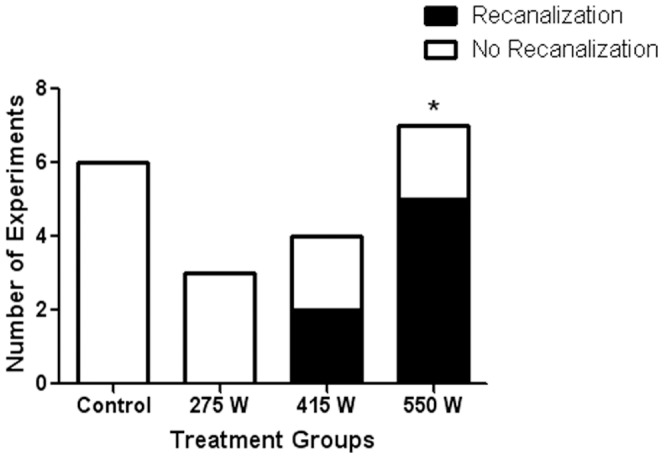
HIFU (550 W) significantly enhances reperfusion. Reperfusion was expressed as a proportion of the total animals tested at each power and the values were compared using Fisher's exact test. HIFU treatment with increasing powers was statistically significant (*p<0.05).

There was no evidence of reperfusion or damage in animals not treated with HIFU (n = 6). The numbers of recanalized arteries are detailed in Table 2. Statistical analysis confirmed that HIFU treatment using 550 W (5/7) was statistically significant compared to non-sonicated controls (0/6) (*p<0.05, Figure 5). The other treatment groups were not statistically different from the control group.

**Table 2:** Summary of recanalization rates following HIFU.

	Recanalization	No Recanalization	Total Animals	Proportion
**Control**	0	6	6	0
**275 W**	0	3	3	0
**415 W**	2	2	4	0.5
**550 W**	5	2	7	0.7

Angiograms taken after clot injection and following HIFU treatment were compared and analyzed. Recanalization was noted if contrast agent was detected in the MCA following the treatment.

## Discussion

In this study we investigated the feasibility of using HIFU as a stand alone method for clot lysis using a rabbit model of embolic stroke. We evaluated several acoustic power levels and observed recanalization of the MCA in 5 of 7 rabbits at the highest power tested. Histological analysis of the region surrounding the targeted vessels indicated that the treated artery remained intact and viable. These results warrant further investigation with a clinically relevant transducer to optimize HIFU as a stand alone method for thrombolysis.

The feasibility of using HIFU to disrupt clots has been previously demonstrated *in vitro* [21], [22] and in peripheral arteries *in vivo* [13], [23]. In these studies, focused ultrasound transducers emitting short, pulsed waves were more effective than continuous wave ultrasound for thrombolysis. The improvement in clot lysis was attributed to cavitation activity induced by the pulsed wave. The effects of cavitation on the brain tissue surrounding the focal spot are relatively unknown. Recently, Maxwell and colleagues [14], [23] described a potential mechanism for their ultrasound pulse sequence which led to controlled cavitation and clot lysis *in vitro* and *in vivo* [14], [23]. By using short pulses (0.05 ms) at >6 MPa peak negative pressure and a pulse repetition frequency (PRF) of 1 kHz, the authors suggest that the formation of a cluster of microbubbles and their subsequent collapse caused local mechanical stress which removed a portion of the targeted tissue. These results are consistent with the work of Wright et al. [13], who, employing 1 ms bursts at 1 Hz PRF, correlated restoration of flow in the femoral artery *in vivo* with an increase in acoustic power and the detection of inertial cavitation. Using the same ultrasound pulse sequence as above but with higher acoustic powers, we confirmed that clot lysis was possible in the rabbit brain and therefore conclude that it is also mediated by inertial cavitation.

In our previous study [13], recanalization in the femoral artery was achieved using 300 W of acoustic power whereas we did not observe recanalization in the MCA until 415 W of acoustic power was used (all other sonication parameters were identical). It has been estimated that the attenuation of ultrasound through ∼2 cm of brain tissue is 5 Np/m/MHz [24], which is similar to the attenuation of skeletal muscle [13], [25]. In the current study, ultrasound is also attenuated by the regenerating skin which is sutured over the cranial window and allowed to heal for 14 days. As a skin wound matures, there is an increase in collagen at the wound site leading to higher ultrasonic attenuation over normal skin [26]. We hypothesize that the regenerating skin further attenuates the ultrasound thereby requiring some increased power for clot lysis, however, this alone is not enough to explain the difference. Another possible reason for the increased power could be the cranial window which, if not large enough, may cause the skull to block part of the beam propagating into the brain. Finally, MRI targeting is not as precise as the visual targeting used by Wright et al. [13], and even a small misalignment could increase the power requirement significantly.

A concern for treatments inducing mechanical thrombolysis is the size of the resulting clot debris as large clot fragments can become emboli and lodge downstream of the blocked vessel leading to further blockages in the capillary bed. *In vitro* analysis has indicated that fragment size is very small with the majority of the debris being <10 µm [13]. Prussian blue was used to detect clot fragments containing iron nanoparticles downstream of the proximal MCA after HIFU treatment. We observed small regions of Prussian blue staining in the cortex of two animals treated with 415 and 550 W, indicating that HIFU mostly releases fragment pieces small enough to be cleared from the cerebral vasculature. Not all of the animals could be assessed for downstream blockages since 5 of the animals in the study received more than 1 clot injection (Table 1). Furthermore, it is possible that blockage of the distal capillary bed was independent of our treatment and due to the unpredictable nature of the embolic stroke model. However, the detection of fragments in the cortex may be significant as it can suggest that HIFU treatment may block portions of the distal capillary bed. If true, we predict that the large fragments detected downstream would be created after a single sonication disrupts the entire clot structure and loosened fragments separate from the main clot. It is hypothesized that rapid scanning over the entire surface of the clot will reduce the production of large fragments. Such rapid scans are feasible using hemispherical phased arrays, which is the prototype design for transcranial HIFU treatments in patients [27]–[29].

In this study, we limited our histological examination to the regions surrounding the focal spot as our main goal was to assess the effects of HIFU on the targeted vessel. We observed bleeding at the base of the brain in some of the animals sonicated at 550 W. In each animal, the recanalized vessels appear to be intact on post-treatment angiograms suggesting that the HIFU treatment does not affect the targeted vessel. CD31 staining confirms that the MCA and other arteries and arterioles in the sonicated region are intact and viable, making the source of blood at the skull base unclear. We speculate that the bleeding is due to the presence of standing waves produced by the interaction of propagating ultrasound waves with waves reflecting off the skull base. Standing waves increase the peak pressure and create regions of high pressure outside of the target area [30], [31]. Furthermore, cavitation events may occur more readily in the presence of standing waves since bubbles migrate toward the standing wave antinodes [31]. We have already reduced the incidence of standing waves from previous studies such as the TRUMBI trial, by choosing to use a high frequency, focused transducer [32]. The 1.5 MHz transducer reduces the size of the focal spot and increases the threshold for cavitation thereby restricting the spatial extent of the induced cavitation even further. However, the focal zone of the transducer is elongated in the axial direction and when targeted to the blockage in the MCA, the focal zone can overlap with the skull base. This proximity translates to reflected waves with higher amplitude and increases the incidence of standing waves. Together, this suggests that the generation of standing waves in these experiments are likely, resulting in greater cavitation and vessel damage. For translation of the current work to clinical practice, it is important to note that the large size of the human brain, with a greater distance between the MCA and the skull, will reduce the incidence of damage. Furthermore, hemispherical transducer arrays are being developed for use in clinical trials. These transducer designs are likely to reduce the presence of standing waves [33] and potential tissue damage. Furthermore, phased arrays are adjusted based on CT maps of bone density to produce a sharp focus through the skull and their large surface area reduces the heating at the skull surface thereby negating the need for a cranial window [34]. A clinical prototype, compatible with MRI, was completed by Insightec Inc (Hafia Israel) [34] and has been used for treatment of brain tumors [35], chronic pain [36] and essential tremor [37]. It is expected that continued improvements to the clinical design will increase the achievable intracranial pressures thereby allowing the device to be used for HIFU treatment of ischemic stroke. The focal spot of current arrays, with frequencies ranging from 650–810 kHz is estimated to be 2×3 mm which will reduce the ultrasound effects on non-targeted tissue and minimize standing waves [28], [33].

As we move towards testing HIFU using a clinically relevant transducer, a complete safety study will be necessary. This includes pre- and post-treatment imaging, functional tests following recovery of the animal and a thorough examination of tissue damage throughout the brain. Sonothrombolysis treatments have produced off-target effects [12] due to the presence of standing waves [32] when unfocused beams have been used. However, the formation of standing waves is much less likely when large area focused arrays are used [33].

There is great need for an alternative treatment for ischemic stroke that promotes clot lysis, reduces the incidence of intracerebral hemorrhage and improves patient outcomes. Our results demonstrate that HIFU, as a stand-alone method, can cause effective thrombolysis and does not damage the targeted vessels. HIFU, combined with imaging modalities used to identify and assess stroke patients, could dramatically reduce the time to achieve flow restoration in patients thereby significantly increasing the number of patients which benefit from thrombolysis treatments.
